# Veterinary Journal

**Published:** 1851-12

**Authors:** 


*Veterinary Journal.* — A *veterinary journal* is issued at Boston, under the editorial charge of George H. Dodd, M. D.

